# Dataset of SSR markers for ISSR-Suppression-PCR to detect genetic variation in *Garcinia mangostana* L. in Peninsular Malaysia

**DOI:** 10.1016/j.dib.2016.08.016

**Published:** 2016-08-16

**Authors:** Sri A’jilah Samsir, Hamidun Bunawan, Choong Chee Yen, Normah Mohd Noor

**Affiliations:** a Institute of Systems Biology, Universiti Kebangsaan Malaysia, 43600 Bangi, Selangor, Malaysia; b School of Environment and Nature Resource Science, Faculty Science and Technology, Universiti Kebangsaan Malaysia, 43600 Bangi, Selangor, Malaysia

**Keywords:** *Garcinia mangostana* L., Genetic variation, DNA markers, Peninsular Malaysia

## Abstract

In this dataset, we present 15 Simple Sequence Repeat (SSR) markers with the motifs (AC)n, (GA)n, and (AC)n(AG)n using a ISSR-Suppression-PCR technique in order to discriminate *Garcinia mangostana* from diverse geographical origins in Peninsular Malaysia. A few loci showed differences between 3 and 6 bp in allele size, indicating that there are some polymorphisms between individuals correlating to the number of SSR repeats that may be useful for differentiate of genotypes. Collectively, these data show that the ISSR-Suppression-PCR is a valuable method to illustrate genetic variation of selected *G. mangostana* in Malaysia.

**Specifications Table**

**Table:** 

Subject area	*Biology*
More specific subject area	*Plant sciences*
Type of data	*Table, figure*
How data was acquired	*Development of molecular markers using ISSR-Suppression-PCR*
Data format	*Analyzed*
Experimental factors	*Three SSR motifs: (AC)n, (GA)n, and (AC)n(AG)n were targeted. PCR amplification was conducted on genomic DNA of Garcinia mangostana to obtain DNA fragments with the SSR motifs at both ends. With this approach, we have developed 15 SSR markers to be used in screening of genetic variation ofG. mangostana collected throughout Peninsular Malaysia.*
Experimental features	*Each of the 15 SSR markers was tested on selected G. mangostana accessions. A few loci showed differences of approximately 3–6* *bp in allele size. These differences showed that there are some polymorphisms between individuals according to the number of SSR repeats.*
Data source location	*Peninsular Malaysia*
Data accessibility	*The data is available with this article.*

**Value of the data**

• These data suggest that ISSR-Suppression-PCR is a useful method to provide information on the genetic variation of selected *G. mangostana* genotypes in Peninsular Malaysia.
• These SSR markers can assist researchers to differentiate between accessions of *G. mangostana*.
• Seven out of 15 SSR markers tested on several accessions of *G. mangostana* showed differences between 3 and 6 bp in allele size revealing genetic variation in this apomictic plant and suggesting the usefulness of these accessions as potential resources for future breeding programs.

## 1 Data

15 SSR markers were developed and tested on selected *Garcinia mangostana* accessions (Table 1) [1]. The data presented here shows that the ISSR-Suppression-PCR technique was very applicable in designing SSR primers and was able to reveal variation of selected germplasm collections [2], [3]. These SSR markers can be used to assess genetic variation of *G. mangostana*.

## 2 Experimental design, materials and methods

### 2.1 DNA extraction

Total genomic DNA was extracted from frozen leaf material of *G. mangostana* using DNeasy Plant Mini Kit (QIAGEN). The purity and concentration of the DNA were estimated using Nanodrop ND-100. The quality of DNA was determined through 1% agarose gel electrophoresis and examined under UV (Fujifilm Intelligent Dark Box Las-3000).

### 2.2 Restricted DNA libraries

Three restriction enzymes: *Alu* I, *EcoR* V and *Hind* III (Promega, USA), were used to digest the genomic DNA with a concentration of 100 ng/μL. The digestion product was analyzed on 1.5% agarose gel. Specific blunt adaptors consisting of a 48-mer (5′-GTA ATA CGA CTC ACT ATA GGG CAC GCG TGG TCG ACG GCC CGG GCT GGT-3′) and an 8-mer with the 3′- end capped by an amino acid residue (5′-ACC AGC CC-NH2-3′) were then used to ligate the fragments. Each adaptor consisted of specific sites for AP1 and AP2 primers. AP1 primer (5′-GTA ATA CGA CTTC ACT ATA GGG C-3′) and ISSR-specific primer IP1, and AP2 primer (5′-ACT ATA GGG ACA CGC GTG GT-3′) and ISSR-specific primer IP2 were used together in primary PCR and secondary nested PCR respectively. The ligation mixture consisted of 21 μL digestion products, 1.6 μL 10× ligation buffer, 1.9 μL 25 μM adaptor and 0.5 μL 6U T4 DNA ligase.

### 2.3 PCR amplification

ISSR-Suppression-PCR was conducted to amplify the ISSR fragment with the SSR motif at each end. Three SSR motifs: (AC)n, (GA)n and (AC)n(AG)n were used in this technique. PCR amplification conditions were the following: initial denaturation for 3 min at 94 °C, followed by denaturation for 40 s at 94 °C; annealing for 45 s at 55 °C; elongation for 1 min at 72 °C and then 35 cycles of 30 s at 94 °C, 45 s at 55 °C and 1 min at 72 °C; and final extension at 72 °C for 10 min. The PCR product was then checked on a 1.5% agarose gel. Amplified fragments of 300–600 bp were excised using a clean razor blade and gel purified using QIAquick Gel Extraction Kit (QIAGEN). The quality of the gel purification product was analyzed using Nanodrop-100 before being used in cloning procedure.

### 2.4 Cloning

The amplified fragments were ligated into a pGEM-T Easy Vector (Promega, USA), following the kit protocol. The ligation products were then transformed into *Escherichia coli* (TOP10). The transformants were spread on an LB plate containing ampicillin, IPTG, and Xgal. The positive clones were selected using a pipette tip, and the tip was rinsed in the PCR cocktail containing: 5 μL 1× PCR buffer, 1 μL 0.2 mM dNTPs, 0.5 μL 10 pmol forward primer (M13F), 0.5 μL 10 pmol reverse primer (M13R), 0.5 μl 2.5 U *Taq* Polymerase, and distilled water was added up to 20 μL. The PCR conditions were the same as described above. The products of the colony PCR were checked on 1.0% agarose gel. Plasmids were isolated from selected positive clones and sent to First Base Sdn. Bhd. (Malaysia) for sequencing. The obtained sequences were edited using BioEdit software (http://www.softpedia.com/get/Science-CAD/BioEdit.shtml*)*. Primer Premier 5 software (http://primer-premier.softpedia.com/) was used to design IP1, IP2, and IP3 primers.

### 2.5 Urea-denaturing PAGE

**Fig. 1:**
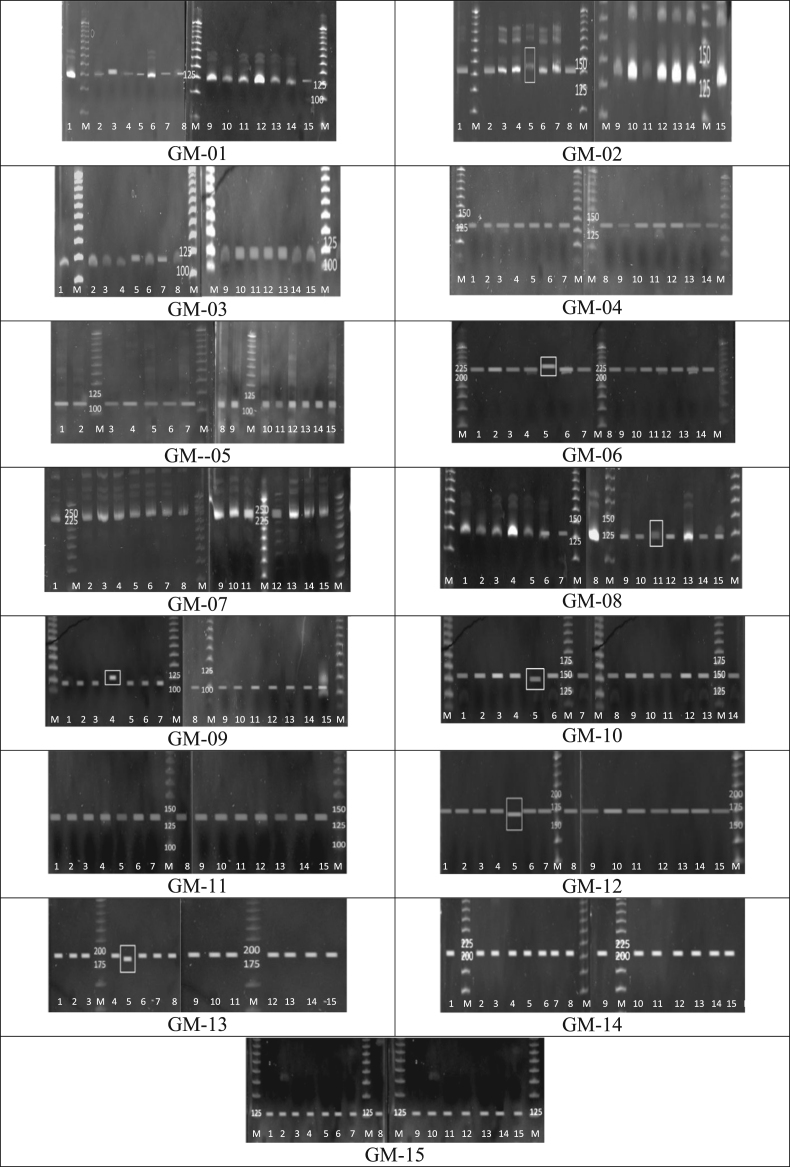
15 SSR markers tested on selected *G. mangostana* accessions [1]. Lanes 1–15: *G. mangostana* accessions from various locations in Peninsular Malaysia. The white box showed differences about 3–6 bp in allele size. M: 25 bp DNA ladder (Promega, USA).

The successfully designed SSR primers (designated as IP2 and IP3) were tested on a few accessions *of G. mangostana* collected from different locations in Peninsular Malaysia (Fig. 1) [1]. The PCR products amplified from these SSR primers were assayed on 8% PAGE gels run for 3 h at 60 V.

## Figures and Tables

**Table 1:** List of IP2 and IP3 primers. Each primer pairs was assigned as one SSR locus.

Primer name	Sequence of IP2 primer (3′–5′)	Sequence of IP3 primer (3′–5′)
GM-01	ACGTGGAGAGCCATCCGAAGT	ATAGATAACGTGAGGGTGGAA
GM-02	TTGAGGAAAAGAAGGTAAACTCTC	ATATTTTGGAATGAAACCTCG
GM-03	TGCCTTTCGGGTGGTGTGTTGTGT	CGCGTGGTGAGCTAAGAAAGT
GM-04	TTCATCTCCTCCTCTTTGACTACT	AACAATTTGAATTGGTTGCCT
GM-05	CTGAAGCCCTCAATTTTCATCTCC	CGACCACTATAGGGACACG
GM-06	GGTGGAGGAAATCCCAACAGTCAG	ATAAAATGATACCCACCTC
GM-07	ATTGGGTACCGGTGGAGAAAA	GAAGCCTATGGGCAACTA
GM-08	TAAATGCCCAAGAAAAGAAGG	TTGGTGAATGAGGGAGCA
GM-09	GTTTAGAAAGTGCTGTGTGAC	GATGTATGGGACCTAATG
GM-10	GACATACAGGAAACGGTGGAG	ATTGTAAATGACCATCAACTA
GM-11	GTCACAACCCAATCTAGGTCG	AGCTAATGGTTTGTAGGGAAA
GM-12	GTAACAACCCAATCTAGGTCG	GCGTGGTGACGGCCACACATT
GM-13	CAGACCATAAGCATGATATGT	AAGGTAAGGACATTATGTG
GM-14	ACCAACTGAGCCTCTGGGCTA	CGAGTTCGCCAACACCTA
GM-15	TTATAAATCAATCGAGCCTTT	TAGAAGCCTACGGGCAAT
