# Supplementary material for: Dataset of SSR markers for ISSR-Suppression-PCR to detect genetic variation in Garcinia mangostana L. in Peninsular Malaysia
Source: Data Brief. 2016 Aug 16;8:1438–42. doi: 10.1016/j.dib.2016.08.016 (PMC5007546; doi:10.1016/j.dib.2016.08.016)
Supplement: Supplementary file 1 — Supplementary material [file mmc1.pdf]

# Conflicts of Interest Statement

**Manuscript title: Dataset of SSR Markers for ISSR-Supression-PCR to detect genetic variation in *Garcinia mangostana* L. in Peninsular Malaysia**

The authors whose names are listed immediately below certify that they have NO affiliations with or involvement in any organization or entity with any financial interest (such as honoraria; educational grants; participation in speakers' bureaus; membership, employment, consultancies, stock ownership, or other equity interest; and expert testimony or patent-licensing arrangements), or non-financial interest (such as personal or professional relationships, affiliations, knowledge or beliefs) in the subject matter or materials discussed in this manuscript.

Author names:

**Sri A'jilah Samsir, Hamidun Bunawan, Choong Chee Yen, Normah Mohd. Noor**

The authors whose names are listed immediately below report the following details of affiliation or involvement in an organization or entity with a financial or non-financial interest in the subject matter or materials discussed in this manuscript. Please specify the nature of the conflict on a separate sheet of paper if the space below is inadequate.

Author names:

**Sri A'jilah Samsir, Hamidun Bunawan, Choong Chee Yen, Normah Mohd. Noor**

rect (*a photocopy of this form may be used if there are more than 10 authors*):

| Author's name (typed) | Author's signature | Date |
| --- | --- | --- |
| Sri A'jilah Samsir | | 1/7/2016 |
| Hamidun Bunawan | | 1/7/2016 |
| Choong Chee Yen | | 1/7/2016 |
| Normah Mohd. Noor | | 1/7/2016 |
| | | |
| | | |
| | | |
| | | |
| | | |
| | | |
